# Chronic Cardiotoxicity Assays Using Human Induced Pluripotent Stem Cell-Derived Cardiomyocytes (hiPSC-CMs)

**DOI:** 10.3390/ijms23063199

**Published:** 2022-03-16

**Authors:** Akshay Narkar, James M. Willard, Ksenia Blinova

**Affiliations:** 1 Center for Devices and Radiological Health, U.S. Food and Drug Administration, Silver Spring, MD 20993, USA; akshay.narkar@fda.hhs.gov; 2 Center for Drug Evaluation and Research, U.S. Food and Drug Administration, Silver Spring, MD 20993, USA; james.willard@fda.hhs.gov

**Keywords:** chronic, cardiotoxicity, cardiomyocytes, electrophysiology, contractility

## Abstract

Cardiomyocytes (CMs) differentiated from human induced pluripotent stem cells (hiPSCs) are increasingly used in cardiac safety assessment, disease modeling and regenerative medicine. A vast majority of cardiotoxicity studies in the past have tested acute effects of compounds and drugs; however, these studies lack information on the morphological or physiological responses that may occur after prolonged exposure to a cardiotoxic compound. In this review, we focus on recent advances in chronic cardiotoxicity assays using hiPSC-CMs. We summarize recently published literature on hiPSC-CMs assays applied to chronic cardiotoxicity induced by anticancer agents, as well as non-cancer classes of drugs, including antibiotics, anti-hepatitis C virus (HCV) and antidiabetic drugs. We then review publications on the implementation of hiPSC-CMs-based assays to investigate the effects of non-pharmaceutical cardiotoxicants, such as environmental chemicals or chronic alcohol consumption. We also highlight studies demonstrating the chronic effects of smoking and implementation of hiPSC-CMs to perform genomic screens and metabolomics-based biomarker assay development. The acceptance and wide implementation of hiPSC-CMs-based assays for chronic cardiotoxicity assessment will require multi-site standardization of assay protocols, chronic cardiac maturity marker reproducibility, time points optimization, minimal cellular variation (commercial vs. lab reprogrammed), stringent and matched controls and close clinical setting resemblance. A comprehensive investigation of long-term repeated exposure-induced effects on both the structure and function of cardiomyocytes can provide mechanistic insights and recapitulate drug and environmental cardiotoxicity.

## 1. Introduction

There have been many advances in basic electrophysiology and translational medicine that have improved our understanding of cardiac function, yet cardiovascular diseases (CVDs) remain the leading cause of mortality worldwide [1]. Studies involving in vitro induced pluripotent stem cell (iPSC)-derived cellular models, animal tissue preparations and in vivo animal models have been developed with an emphasis on experimental utility and clinical relevance [2,3,4,5,6,7]. Functional hiPSC-derived cardiomyocytes (CMs) have been used as a model to study various cardiac diseases, such as dilated cardiomyopathy [8], long-QT syndromes [9,10], cardiac hypertrophy [11] and catecholaminergic polymorphic ventricular tachycardia [12,13]. hiPSC-CMs offer a novel in vitro platform for pre-clinical cardiotoxicity and pro-arrhythmia screening of drugs in development [14,15,16,17,18]. One of the important advantages of the current iPSC-CMs as compared to scarcely available primary heart cells is the ability to culture and study them for long periods of time (days to weeks, at least). Some drugs including, but not limited to, hERG channel trafficking (pentamidine) and chemotherapeutic (doxorubicin) [19,20,21,22,23] can cause an accumulation of the compound in the body when an acute dose may not be toxic but, due to chronic (several hours to days) exposure, may become toxic due to prolonged exposure or the delayed effects of drugs. Cardiac complications that occur late after therapy are missed in pre-clinical studies focusing on acute time points, ranging from seconds to a few hours after administration. From a clinical point of view, it is critical to understand delayed onset, and thus, there is a pressing need to standardize long-term assays for detecting cardiotoxicity, both structural and functional aspects, in a pre-clinical setting.

Cardiotoxic side effects of drug therapies have been well established since the 1940s, with treatment of local anesthetics and digitalis having an adverse effect on cardiac function [24]. In 2019, a scientific statement on behalf of the American Heart Association Council on Basic Cardiovascular Sciences was issued [25], highlighting the unique role of hiPSC-CMs in the assessment of chronic cardiotoxicities induced by cancer treatments. The statement that reviewed 100+ scientific publications named reproducibility as one of the main challenges in the development of hiPSC-CM-based assays for cardiotoxicity assessment. Indeed, while commercially available iPSC-CMs manufactured at a large scale promote reproducibility, the high cost of these cells might be prohibitive for some laboratories. According to the authors, the establishment of reproducible cardiotoxicity assays relies on accurate and comprehensive information on hiPSC-CMs generation protocols, adoption of new advanced techniques and use of the cells with standardized protocols, following accepted best practices. The review characterized drug-induced cardiotoxicity into three broad categories: electrophysiology, contractility and injury/structural damage and discusses the common biomarkers to quantify each along with the technological approaches’ advantages and disadvantages. For example, measuring drug-induced changes in hiPSC-CM action potential using voltage-sensitive dye (a common biomarker for electrophysiology toxicity) has an advantage of the direct visualization of drug effects on the cellular action potential shape, suggesting effects on the specific ion channels. However, care should be taken when interpreting results since the expression of key ion channels in hiPSC-CMs is known to differ from primary human adult cardiomyocytes. The AHA statement concludes with the final remark on hiPSC-CMs’ potential to lead the way in the assessment of direct cardiotoxic effects of oncological agents in the near future.

**Figure 1:**
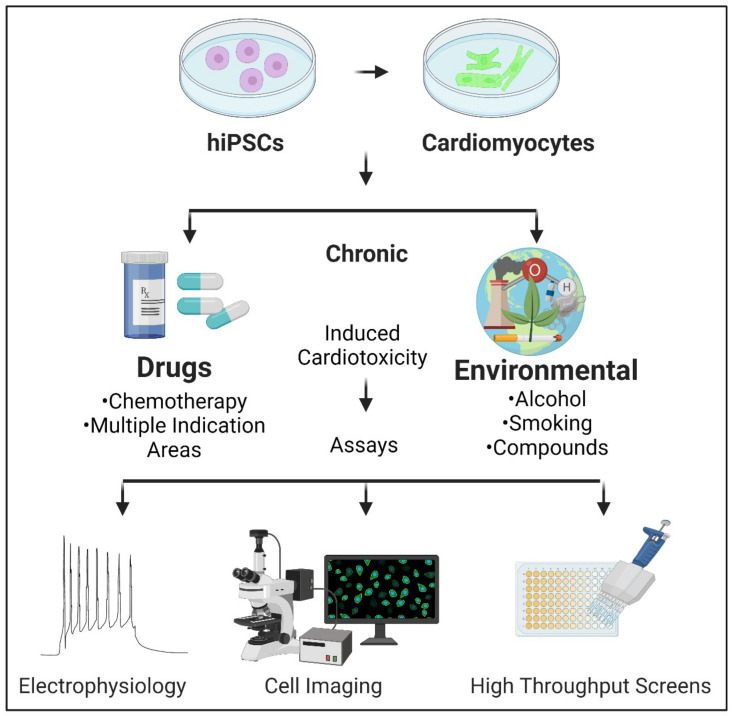
Cardiotoxicity screening using human induced pluripotent stem cell (hiPSC) derived cardiomyocytes post prolonged exposures.

In this review, we focus on the most recently reported chronic cardiotoxicity assays using hiPSC-CMs (Figure 1 and Table 1). Observations from chronic studies performed on cardiomyocytes using drug candidates from indication areas such as anti-cancer, antibiotics, anti-hepatitis C virus (HCV) and antidiabetic agents are discussed. In addition, we highlight recent extended applications of hiPSC-CMs to study the long-term effects of alcohol consumption and environmental compound exposure on hiPSC-CMs in a chronic setting.

## 2. Assessing Chemotherapy-Induced Chronic Cardiotoxicity Using hiPSC-CMs

During the past few years, although chemotherapies have improved long-term survival rates, there is, unfortunately, an additional increased risk of cardiac complications because of underlying long-term adverse effects of these anti-cancer agents [42,43,44]. The primary application area of hiPSC-CM assays has focused on cardiotoxicity screening of several anti-cancer agents [45,46,47,48,49,50]. In the last several decades, there have been tremendous advances in anticancer drug discovery and research, with more than 100 anticancer drugs that have been approved by the FDA [51,52,53]. Since its discovery in the early 1960s, anthracycline regimens have been the mainstay of chemotherapy [20]. Data indicate that anthracycline-induced (ROS) reactive oxygen species may be dependent on topoisomerase-2β [54]. Thus, of particular interest are chemotherapy agents, such as doxorubicin, a known inhibitor of DNA enzyme topo isomerase 2, to induce growth arrest [55]. Chronic doxorubicin-induced cardiomyopathy has an incidence ranging from 4–36% based on dose and can occur as late as 10 years after last dose, making it extremely challenging to predict in acute studies [56,57]. Sadly, there is only a 50% 1 year survival prognosis when cardiomyopathy develops into congestive heart failure [58,59,60,61].

To investigate genomic biomarkers for cardiotoxicity with repeated chronic exposure of anthracycline family members, including doxorubicin [26], Chaudhari et al. used commercially available hiPSC-CMs (iCell Cardiomyocytes^2^, Cellular Dynamics). Cells were incubated with 156 nM doxorubicin for 2 or 6 days, followed by washout in compound-free culture medium until day 14 after the onset of exposure. Endpoint assays included cytotoxicity and beating frequency of hiPSC-CMs, using a xCELLigence Real Time Cell Analyser (ACEA Biosciences). In contrast to a single exposure of doxorubicin, repeated exposure caused long-term arrhythmic beating and cytotoxicity. The study also analyzed transcriptomic data to identify a set of genes involving a sarcomeric structure, apoptosis regulators and genes modulating ion homeostasis. Further validation was performed by real-time PCR using 10 nM danorubicin, 3 nM mitoxantrone and 156 nM doxorubicin for a 48 h exposure to hiPSC-CMs. This long-term study indicates that the commonly downregulated 27 genes and upregulated 8 genes can be potentially applied as a gene panel for a safety assessment of novel drug candidates. Chronic hiPSC-CMs assays have also been developed for the screening of potential cardioprotective candidates that could decrease anticancer drug-induced cardiotoxicity [62]. The effects of doxorubicin with or without the cardioprotective agent were measured for up to 3 weeks using the standard cell viability assay based on tetrazolium dye MTT 3-(4,5-dimethylthiazol-2-yl)-2,5-diphenyltetrazolium bromide. The hiPSC-CMs experiments were paralleled by experiments in primary neonatal rat ventricular myocytes. The authors emphasized the importance of an appropriate experimental model for compound testing in early drug development, as illustrated by their study where the delayed toxicity of the compound under investigation was only evident in hiPSC-CMs but not in the primary animal cardiomyocytes due to the significant differences in the duration of the experiments supported by each model.

Histone deacetylase HDACs are regulatory enzymes that remodel epigenetic chromatin structure and are widely used to treat certain cancers, autoimmune and neurological diseases. Kopljar et al. used transcriptional and functional assays to study cardiotoxicity towards histone deacetylase (HDAC) inhibitors using cardiomyocytes [27]. This study subjected Cor.4U CMs from Axiogenesis to HDAC inhibitors, such as dacinostat, panobinostat, vorinostat, entinostat and tubastatin A in a chronic setting to follow the long-term effects of HDAC inhibitors on the beating properties of Cor.4U-CMs using an impedance-based functional assay. Impedance signals were recorded using the xCELLigence Cardio instrument (ACEA Biosciences-Roche Diagnostics) at baseline and after a compound addition at ≤1 h intervals during 84 h, and multi electrode assay (MEA) experiments were recorded at baseline and 6 and 24 h after dose. Post HDAC inhibitor treatments observations included contractile dysfunction, arrhythmic events and shortening of field potential duration (FPD). Transcriptional responses based on (2, 4 h) and (12, 24 h) were used to investigate affected gene sets. At 12 h, signaling pathways regulating microtubule and cytoskeleton transport, cardiac contractility and unfolded protein binding seemed to be differentially expressed. This opens up avenues to explore epigenetic modifications of the downstream mechanistic effects that are markers for certain type of cardiovascular disease risk factors.

Another class of compounds commonly used as anti-cancer therapeutics are Tyrosine kinase inhibitors (TKIs). Sharma et al. [28] investigated the cardiotoxicity of 21 FDA-approved TKIs using patient-specific hiPSC-CMs, reprogrammed in the lab using published protocols from 11 healthy individuals and 2 patients receiving TKI cancer treatment. Cells were treated with TKIs at ranges 0–100 µM for 72 h in most cases, and the assay endpoints included cardiomyocytes viability, kinase phosphorylation profiling, contractility and signaling. Vascular endothelial and platelet-derived growth factor receptor (VEGFR2/PDGFR) inhibiting TKIs, such as sorafenib, regorafenib and ponatinib, demonstrated significant cytotoxicity in hiPSC-CMs, with LD50 of 3.4 μM, 7.1 μM and 4.3 μM, respectively. The study also performed a contractility assessment in commercially available hiPSC-CMs to minimize variability. The analysis of effective concentration, cessation of beating in vitro, cytotoxicity, LD50 data and normalization to literature reported Cmax values in which the group developed a “cardiac safety index” as a metric to screen cardiotoxic TKIs. The study also sheds light on a model for activation of compensatory cardioprotective insulin/IGF signaling in hiPSC-CMs in response to TKIs of VEGF pathway. Other studies have performed chronic assays using hiPSC-CMs to investigate metabolite signatures of doxorubicin-induced toxicity [23], contractile motion properties and cardiac biomarker development [63] and demonstrated that continued exposure to anticancer drugs can unmask their cardiotoxic effects [64]. As presented above, a combination of electrophysiological, functional, structural and genetic assays with long-term exposure can enable the development of efficient high-throughput hiPSC-CMs platforms for pre-clinical chemotherapy-induced cardiotoxicity screening.

## 3. Non-Cancer Drug-Induced Cardiotoxicity Screening Using hiPSC-CMs

Drug classes including, but not limited to, psychostimulants, analgesics, anesthetic, antihistamines, propulsive, antimicrobial, antiparkinsonian, emetic and bronchodilators can demonstrate cardiovascular adverse events [65]. Safety pharmacology studies for promising new drug candidates may include a broad panel of agents for assessing cardiotoxic effects. A large study by Doherty et al. [29] used commercially available hiPSC-CMs (iCell Cardiomyocytes) to test 24 drugs from multiple indications with known clinical cardiac risks. Drug types included antibiotics, chemotherapy agents, antihistamines, anti-arrhythmics, NSAID, etc. Recordings (30 min interval) were then taken at 24 h and 48 h to assess time-dependent drug impacts. A structural assessment included reactive oxygen species (ROS) generation, viability, troponin secretion and lipid formation and functional characterization, focused on beating activity as the endpoint. In contrast to cardiac-safe drugs, 16 of 18 compounds with known cardiac risks showed drug-induced changes in hiPSC-CMs in one or more assays. An important consideration while refining in vitro safety pharmacology studies, particularly cardiotoxicity, are a combination of both structural and functional endpoints. The structural damage at the cellular and tissue level may take longer to manifest depending on the stage of development, tissue architecture and environment. This study highlights how both these endpoints can provide a more comprehensive assessment of drug-induced cardiac risk in a chronic setting across multiple therapeutic indications and that care must be taken while interpreting the results, as sensitivity and specificity of endpoints can vary across instruments and sites.

Delayed effects of drugs can last for several months in the clinic. Pabel et al. [30] recently studied the long-term effects (up to 2 months) of empagliflozin, an antidiabetic drug, on excitation–contraction (EC) coupling, using laboratory reprogrammed hiPSC-CMs. Four- to 5-week-aged hiPSC-CMs were treated with 0.5 μmol/L empagliflozin for 2 or 8 consecutive weeks, and endpoints included action potential measurements, calcium transients and RNA sequencing analysis. Empagliflozin did not show any significant effects on cardiomyocytes Ca^2+^ transients, sarcoplasmic Ca^2+^ load and diastolic sarcoplasmic Ca^2+^ leak. Action potential was not changed post-chronic empagliflozin treatment when investigated with patch-clamp experiments. Immunoblotting and RNA seq analysis did not show differential expression of EC coupling candidates. Based on the data, the authors concluded that improved cardiovascular outcomes in diabetic patients with chronic empagliflozin treatments are likely independent of EC coupling mechanisms. Another recent study by Yanagida et al. [31] investigated cardiac contractility post-chronic exposure to BMS-986094—an anti-hepatitis C virus (HCV) drug candidate—alongside sofosbuvir, another nucleoside analog inhibitor of HCV. The authors show that long-term drug treatments (≥4 days) with BMS-986094 induced the contraction dysfunction in iCell Cardiomyocytes^2^ at 0.3–3 µM, while sofosbuvir had little effect. Video microscopy for motion vector analysis revealed that BMS-986094 treatment decreased the calcium transient and inhibited the expression of calcium handling-related genes. These studies highlight the application of hiPSC-CMs to investigate clinically relevant chronic effects (up to 2 months) of empagliflozin on cardiomyocyte (EC) coupling and changes in contraction parameters post long-term exposure with HCV drug candidate.

Another area of hiPSC-CMs long-term application is drug target validation. The importance of chronic assay for drug target validation is paramount, as several lead molecules that pass the early safety screening are later (late phase clinical trials or post marketing) withdrawn, owing to cardiotoxic side effects. A study from Fiedler et al. [32] investigated the effects of MAP4K4 inhibition on hiPSC-CMs survival and target validation. Human iPSC-CMs were obtained from commercial sources, such as Cellular Dynamics (iCell CM) and Axiogenesis (vCor.4U), for shRNA knockdown and inhibitor assays. Assay endpoints included contractile properties, calcium transients and mitochondrial assays. vCor.4U ventricular myocytes were assayed 24 h after oxidative stress, conferred by H_2_O_2_ or menadione at the indicated concentration ranges, 0–15 µM, for 72 h, which was countered by F1386-0303, a potent MAP4K4 inhibitor demonstrating protection against lethal oxidative stress. The authors also provided data to demonstrate that MAP4K4 inhibition rescues mitochondrial function and contractile function over chronic time points. This study demonstrates how narrowing the target validation window can significantly assist the current cardiac drug discovery pipeline. hiPSC-CMs act as a model platform for gene silencing and drug screening that are accessible, scalable and can be monitored over several days to weeks to observe changes in morphology, electrophysiology and metabolism. Long-term changes in metabolism can alter the protein and cellular response of cardiomyocytes. Palmer et al. [33] recently developed a metabolic biomarker-based assay that predicts the cardiotoxicity potential of compounds, based on changes in the metabolism and viability of commercially available hiPSC-CMs (iCell CM^2^). Ultra-performance liquid chromatography-high-resolution mass spectrometry (UPLC-HRMS) was used to profile the metabolic response following drug exposure for 72 h. From the viability data and metabolomics screen, four metabolites from pathways for arachidonic acid, lactic acid, 2′-deoxycytidine and thymidine were identified as indicators of cardiotoxicity. The authors demonstrated metabolic profiling experiments and predictive modelling as complimentary alternatives for identifying structural and functional cardiotoxicants. This opens up new avenues using a metabolomics footprint as measured in media of cell culture, reflecting changes in the metabolism of hiPSC-CMs as an evaluation criterion for chronic toxicity studies. Another large international multisite study is underway, spearheaded by the Health and Environmental Sciences Institute (HESI), undertaking the daunting task of testing the effects of several compounds on hiPSC-CMs in a chronic setting. The goal is to not only study electrophysiology, but also investigate the effects on structure, contractility, biomarker release and metabolic activity to better understand molecular signatures and chronic cardiotoxicity.

## 4. Moving beyond Drug-Induced Cardiotoxicity

The use of hiPSC-CMs-based chronic assays is being extended beyond drug testing. Animal models used to establish a link between deleterious alcohol effects and cardiac malformations have limitations both in species physiology and high throughput screening ability. In this study [34], the model was used to mimic chronic alcohol consumption. hiPSC-CMs generated in the lab by directed differentiation were treated with clinically relevant doses of ethanol for up to 5 weeks. Results demonstrated a range of cardiotoxic effects, including cell death, oxidative stress, altered Ca^2+^ handling and abnormal action potential and contractility. Another study by Rampoldi et al. [35] exposed hiPSC-CMs to various doses of ethanol (0, 17, 50 and 100 mM ethanol) for 5 days. They demonstrated chronic ethanol exposure reduces cell viability, generates cellular oxidative stress and increases production of reactive oxygen species (ROS). Additionally, ethanol exposure also generated dose-dependent increased irregular Ca^2+^ transients and contractility in hiPSC-CMs. These results suggest that hiPSC-CMs can be a novel and physiologically relevant system for the study of chronic alcohol-induced cardiac toxicity. However, additional studies are needed to determine the reproducibility and consistency of long-term studies testing ethanol-induced cardiotoxicity, including both structural and functional endpoints. Like alcohol, nicotine can be detrimental to cardiac development [36,66,67]. Cigarette smoking can impair cardiac development and function, yet mechanistic details of nicotine exposure remain elusive. A study from Guo et al. [68] used hESC-CMs and exposed them to nicotine in a chronic setting, extending up to several days. Nicotine exposure altered the transcriptome of hESC differentiation, as demonstrated by single cell RNA sequencing. Long-term exposure of nicotine reduced hESC-CMs cell viability, increased ROS and Ca^2+^ signaling was affected, increasing the risk of arrhythmias. These results are further extended by Basma et al. [37], where they exposed hiPSC-CMs (iCell CMs) to electronic cigarette extract (ECE) and conventional cigarette smoke extract (CSE) for acute and chronic (14 days) exposures. Both ECE and CSE generate ROS, cytotoxicity and slowed beating. Chronic smoking and alcohol exposure are important risk factors for cardiac diseases. The above-mentioned studies can help understand the potential underlying molecular mechanisms, which are difficult to decipher in vivo due to difficulties in obtaining heart tissue from patients.

Another example of hiPSC-CMs assay being used to study cardiotoxicity induced by a non-drug compound is the sub-chronic (12–24 h) exposure of dichlorodiphenyltrichloroethane (DDT), an insecticide that was used in agriculture until its ban in 1972 but is still ubiquitously found in the environment and associated with cardiovascular disease, which was studied in hiPSC-CMs (iCell CM^2^) [38]. Exposure to environmental chemicals and compounds such as Perfluorooctane sulfonate and bisphenol A (BPA) demonstrated mitochondrial damage and altered hESC-CMs morphology and cardiac gene expression [39,40,41] post long-term exposure. Our laboratory is currently developing a chronic hiPSC-CMs-based assay to be applied to the safety evaluation of the implantable medical devices that are designed to improve cardiac contractility in heart failure patients. Cardiac contractility modulation (CCM) is an intracardiac therapy by means of which non-excitatory electrical simulations are timed to coincide with the absolute refractory period. This work is based on the recently developed hiPSC-CM assay [69] to assess the acute effects of cardiac contractility modulation (CCM) therapy. This study demonstrates the application of the hiPSC-CMs as an in vitro human CCM model and reports that CCM increases contractility and modifies calcium handling properties of hiPSC-CMs. Other CCM effects included shortening of action potential duration and increased myofilament calcium sensitivity. These studies demonstrate the implementation of stem cell-derived cardiomyocytes as a novel platform to assess long-term CCM therapy and industrial and environmental compound-induced cardiotoxicity in a chronic setting.

Lastly, an exciting avenue to use hiPSC-CMs as an in vitro model is to perform comprehensive genome wide screens. Diez-Cunado et al. [70] performed a genome-wide miR’ome screen (~875 synthetic human miRNA mimics) to identify miRNAs that promote hiPSC-CMs proliferation. The group used commercial hiPSC-CMs (iCell CM) to perform a primary screen for DNA duplication using 5-ethynyl-2′-deoxyuridine (EdU) and sarcomeric α actinin staining, followed by secondary screen targeting cytokinesis and DNA duplication in hiPSC-CMs. The primary and confirmatory screen yielded ~127 miRNAs that can promote DNA synthesis, and the secondary screen revealed ~96 miRNAs that can modulate cell division. Using computational and chemical screening with 160 small molecule kinase inhibitors, the authors filtered candidates relevant to hiPSC-CM proliferation. This additional testing narrowed down the potential pathways to ~8. Rescue experiments with siRNA demonstrated Hippo/YAP as an important regulator of basal proliferation rate. The study also carefully investigated the essentiality of individual miRNAs towards proliferation by knockdown. The results shed light on a mechanistic model, where multiple endogenous miRNAs redundantly suppress the Hippo pathway to sustain the cell cycle of hiPSC-CMs. Genome engineering is a rapidly evolving field, and long-term assays using hiPSC-CMs can be used to study cardiac development and test the effects of genetic manipulation for better disease modeling and targeted therapeutics.

## 5. Discussion

**Figure 2:**
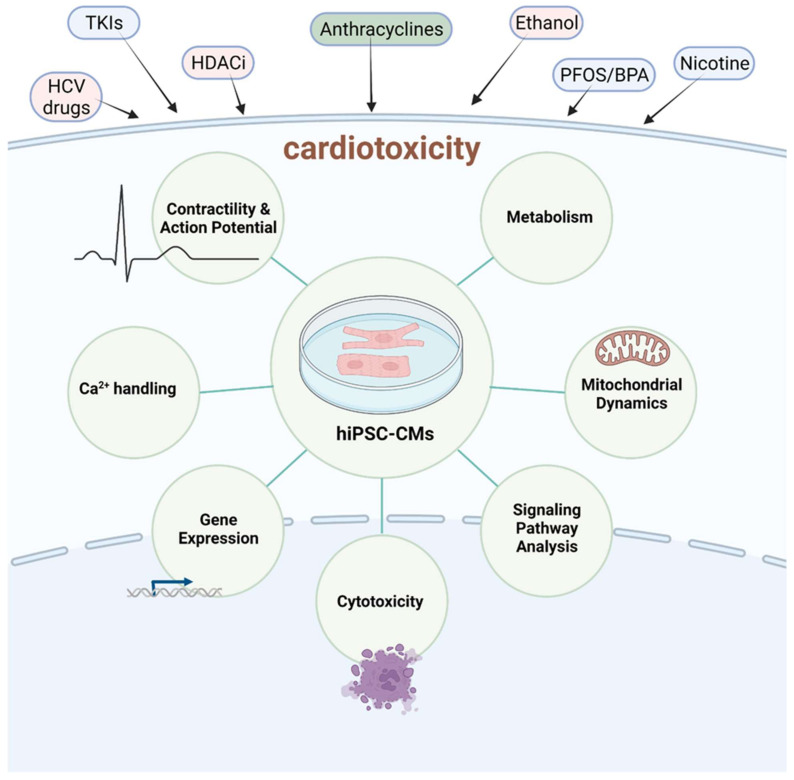
Potential cardiotoxicity mechanisms and chronic assay endpoints.

**Figure 3:**
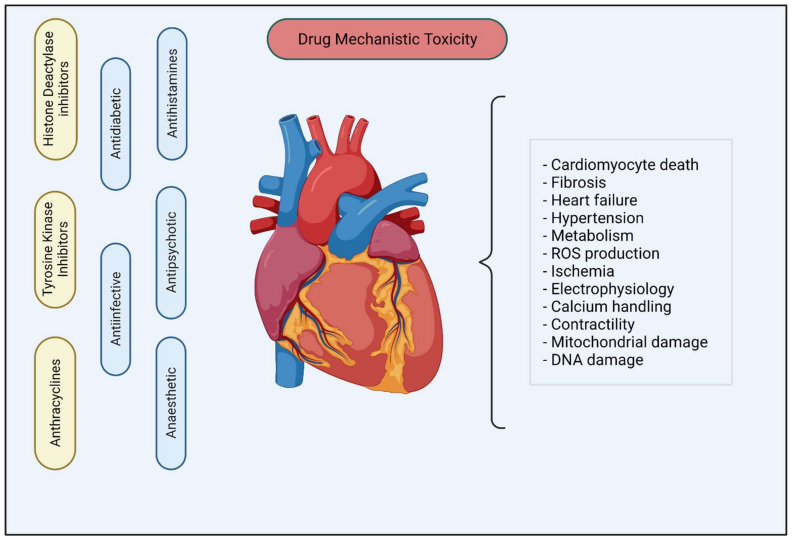
Mechanistic toxicity of anti-cancer drugs and drugs belonging to other indication areas.

There has been tremendous growth in cardiovascular research; this has been fueled by the implementation of hiPSC-CMs as an in vitro model to study drug toxicity, disease modeling and safety assessment. A significant number of hiPSC-CMs assay-based studies focused on acute responses and time points and thus may not recapitulate the electrophysiology and cardiomyocytes responses after prolonged exposure. This review highlights the most recent advances in chronic cardiotoxicity assays using hiPSC-CMs to address this concern (Table 1 and Table 2). Observations from studies with chemotherapeutic agents such as doxorubicin, mitoxantrone, HDAC inhibitors and TKIs demonstrate that, in contrast to single exposures, repeated long-term exposures modulate gene expression, arrhythmic beating, contractile dysfunction and cytotoxicity. These studies also shed light on better understanding the underlying molecular mechanisms in hiPSC-CMs, such as the activation of compensatory cardioprotective insulin/IGF signaling in response to TKIs of the VEGF pathway, and MAP4K4 inhibition rescues mitochondrial and contractile function over chronic time points. Drugs belonging to other indication areas such as anti-histamines, antibiotics, antidiabetics and antivirals may exhibit minimal or significant changes in cardiomyocyte EC coupling, contractility and gene expression (Figure 2 and Figure 3). A long-term metabolomics screen using hiPSC-CMs can help identify novel metabolites and signaling pathways that can act as potential indicators for cardiotoxicity screening. We also discuss the implementation of hiPSC-CMs to study the effects of long-term exposure of clinically relevant doses of ethanol, which elicits cardiotoxic effects, such as cell death, oxidative stress, altered Ca^2+^ handling and abnormal action potential. Other environmental and industrial compounds, such as DDT, nicotine, BPA, etc., alter cardiomyocyte morphology, mitochondrial function and transcriptomic changes (Figure 2).

Key advantages of hiPSC-CMs [71,72,73,74] that have played a critical role in disease modeling and drug development include, but are not limited to, high throughput potential, non-invasive and easy to obtain large numbers, presence of major cardiac ion channels, calcium release and functional sarcoplasmic reticulum calcium stores present robust cardiac currents, such as INa, IKr, ICa, etc. Although these chronic assays provide novel insights into delayed signaling pathways related to cardiotoxicity from a variety of upstream insults, one needs to keep in mind that hiPSC-CMs still remain a simplified in vitro model of the human heart. There are certain limitations of hiPSC-CMs that include: hiPSC-CMs exhibit more circular cell shape, smaller size and mononuclear morphology as compared to adult CMs, which are larger and more elongated and show a higher percentage of binuclear cells. Changes in myocardial metabolism are significant during fetal to adult development. Metabolism in hiPSC-CMs can be dependent on glucose/glycolysis (lab differentiation protocols) or in some commercially available cell lines, which may shift towards fatty acid oxidation, whereas adult CMs predominantly undergo fatty acid/ β-oxidation. Using an ultra-structure analysis, it was observed that hiPSC-CMs exhibit differences in sarcomere length and mitochondrial structure, and T-tubules are absent as compared to adult CMs. A mixture of atrial and ventricular myocytes can demonstrate differential expression of structural genes, such as myosin heavy chain 7 (MYH7) and sarcoplasmic reticulum ATPase (SERCA2). Electrophysiological properties, such as upstroke velocity, spontaneous beat rate, resting membrane potential and conduction velocity, should be carefully investigated before compound testing to achieve comparable baselines. Caution must be exercised while reprogramming iPSCs in the lab, and also details such as donor type, batch/lot number and media supplements must be taken into account when purchased from commercial vendors to achieve reproducibility across sites and minimize variations [75]. Factors such as maturity, cellular interactions, co-culture with non-cardiac cells [70,71,72,73,74] and 3D tissue architecture [75,76,77] are limitations that are being addressed by commercial vendors and research laboratories, and strategies are being implemented to eventually overcome the majority of these hurdles to produce clinical grade cardiomyocytes.

In the most recent year’s studies, there have been significant advances in commercial cardiomyocyte production, 3D assay development [76,77,78,79] and refinement of CRISPR-Cas9 mediated gene editing [80,81,82,83] that will help overcome a majority of these current limitations. Further research is warranted to standardize these long-term, high-throughput assay conditions, improving the reproducibility and sensitivity of endpoints to allow for translational therapeutics. Overall, the studies reviewed here demonstrate the growing acceptance and adoption of using hiPSC-CMs in a chronic setting to facilitate repeated drug exposure and compound screening, providing novel mechanistic insights into delayed signaling and shaping regulatory standards.

## Figures and Tables

**Table 1:** Summary of drugs/compounds, dosage and timepoints tested on hiPSC-CMs along with key findings.

Drugs/Compounds	Time Points	Dosage	Cells	Assays	Highlights/Findings	Reference
Doxorubicin (Other anthracyclines tested)	2 or 6 days followed by washout	156 nM; variable for other anthracyclines	hiPSC-CMs (iCell Cardiomyocytes^2^, Cellular Dynamics)	Cytotoxicity and beating frequency; xCELLigence Real Time Cell Analyser (ACEA Biosciences)	Repeated exposure caused long-term arrhythmic beating and cytotoxicity.	[26]
Histone deacetylase (HDAC) inhibitors, (dacinostat, panobinostat, vorinostat, entinostat and tubastatin-a)	Variable, range 0–80 h assay dependent	Variable, range: 10 nM–10 µM	Cor.4U CMs from Axiogenesis	Impedance signals were recorded using the xCELLigence Cardio instrument (ACEA Bio-sciences-Roche Diagnostics) at baseline and after compound addition at ≤1 h intervals during 84 h, and multi electrode assay (MEA) experiments were recorded at baseline and 6 and 24 h after dose. Transcriptional responses based on (2, 4 h) and (12, 24 h) were used to investigate affected gene sets.	Contractile dysfunction, arrhythmic events and shortening of field potential duration (FPD).	[27]
Tyrosine kinase inhibitors (TKIs), 21 compounds	72 h in most cases	0–100 µM in most cases	Patient-specific hiPSC-CMs, reprogrammed in the lab; also used commercially available hiPSC-CMs.	Cardiomyocytes viability, kinase phosphorylation profiling, contractility and signaling.	Developed a “cardiac safety index” as a metric to screen cardiotoxic TKIs. Molecular model for activation of compensatory cardioprotective insulin/IGF signaling in hiPSC-CMs in response to TKIs of VEGF pathway.	[28]
Multiple indications; antibiotics, chemotherapy agents, antihistamines, anti-arrhythmics, NSAID, etc. (24 drugs)	30 min−48 h	Variable, range: 0.01–100 µM	Commercially available hiPSC-CMs (iCell Cardiomyocytes)	Structural assessment included reactive oxygen species (ROS) generation, viability, troponin secretion and lipid formation and functional characterization focused on beating activity.	Combination of both structural and functional endpoints to better assess drug induced cardiac risk.	[29]
Empagliflozin	2 or 8 weeks	0.5 μmol/L	Laboratory reprogrammed hiPSC-CMs	Action potential measurements, calcium transients and RNA sequencing analysis.	Improved cardiovascular outcomes in diabetic patients with chronic empagliflozin treatments are likely independent of EC-coupling mechanisms.	[30]
BMS-986094, sofosbuvir	≥4 days	0.3–3 µM	iCell Cardiomyocytes^2^ commercial	Video microscopy for motion vector analysis.	Decreased calcium transient and inhibited the expression of calcium handling-related gene.	[31]
H_2_O_2_ or menadione	Assay dependent Range 0–72 h	0–15 µM	Commercial sources, such as Cellular Dynamics (iCell CM) and Axiogenesis (vCor.4U)	Contractile properties, calcium transients and mitochondrial assays.	MAP4K4 inhibition rescues mitochondrial function and contractile function over chronic time points.	[32]
Multiple indications, (81 drugs)	72 h	0.03–100 µM	commercially available hiPSC-CMs (iCell CM^2^)	Ultra-performance liquid chromatography-high-resolution mass spectrometry (UPLC-HRMS) was used to profile the metabolic response.	Four metabolites from pathways for arachidonic acid, lactic acid, 2′-deoxycytidine and thymidine were identified as indicators of cardiotoxicity.	[33]
Ethanol	Variable: 5 days–5 weeks	0, 17, 50, 100, 200 mM	hiPSC line IMR90 (WiCell Research Institute), lab differentiated	Contractility, action potential, viability, calcium handling.	Dose-dependent increased irregular Ca^2+^ transients and contractility. Reduces cell viability, generates cellular oxidative stress.	[34,35]
Nicotine, electronic cigarette extract (ECE) and conventional cigarette smoke extract (CSE)	0, 14, 21 and up to 28 days	1, 10 µM and 0–10% extract	hESC-CMs, hiPSC-CMs (iCell CMs)	RNA seq., cytotoxicity, beat rate analysis.	Altered transcriptome, reduced cell viability, increased ROS and Ca^2+^ signaling was affected.	[36,37]
Dichlorodiphenyltrichloroethane (DDT), Perfluorooctane sulfonate (PFOS) and bisphenol A (BPA)	Variable; range: 0–14 days	0–10 µM, PFOS 0–75 µg/mL, BPA (8 ng/mL)	hiPSC-CMs (iCell CM^2^), hESC-CMs	Contractility, calcium handling, cytotoxicity.	Altered calcium oscillation frequency and storage. Mitochondrial damage, altered hESC-CMs morphology and cardiac gene expression, cardiomyocyte hypertrophy.	[38,39,40,41]

**Table 2:** List of potential chronic cardiotoxicity assays.

Endpoint	Experimental Approaches	Comments
Contractility	Optical imaging, direct contractility force measurement, impedance (cell attachment).	Direct measurements of hiPSC-CMs contractility force can be technically difficult and low-throughput. Indirect approaches, as optical imaging or impedance measurements, may be used as a surrogate of contractility force.
Electrophysiology	Voltage sensitive fluorescent probes, microelectrode array recordings.	Ratiometric voltage-sensitive fluorescent probes account for motion artifacts but might affect cellular physiology, especially in chronic recordings; microelectrode array recordings are probe-free and high-throughput, but field potential data interpretation might be more complicated.
Intracellular calcium transients	Calcium-sensitive fluorescent probes used either in low- or high-throughput assays.	Calcium-sensitive probes might also affect cellular physiology. These probes are relatively bright when compared to voltage-sensitive dyes.
Cell metabolism	Biomarker panels, mitochondrial membrane potential, mitochondrial metabolism (i.e., oxygen consumption rate)	Includes lactic acid, arachidonic acid, thymidine. Measurements based on spent cell culture medium, thus minimally invasive. Metabolism can be significantly altered, depending on the levels of glucose/fatty acids in chronic assay medium.
Cell morphology	Hypertrophy, cell size, myofilament deposition, structural toxicity, fluorescent sarcomere organization	hiPSC-CM are significantly morphologically different from primary human cardiomyocytes, and cell morphology change interpretation might be difficult. Best used in combination with functional assays.
Genetic analysis	Quantitative real time PCR (polymerase chain reaction) assays, next generation sequencing	It is now feasible to analyze transcriptomes at the single-cell level within heterogeneous cell populations. A terminal assay (can only be done once on each cell population), as it requires nucleic acid extraction.
Cell death	Apoptosis—caspase activity, annexin V, propidium iodide staining	Invasive since dyes may penetrate over time, depending on membrane integrity, nuclear pore permeability. Biochemistry may require lysis for protein extraction.
